# A robust binary secretary bird optimization method for high-dimensional data classification

**DOI:** 10.1038/s41598-026-57577-0

**Published:** 2026-07-10

**Authors:** Reham Kamal, Eman Amin, Diaa Salama AbdElminaam, Rasha Ismail

**Affiliations:** 1 Faculty of Computer and Information Sciences, Ain Shams University, Cairo, Egypt; 2 Faculty of Computer Science, Misr International University, Cairo, Egypt; 3 Faculty of Computer Science and Artificial Intelligence, Benha University, Benha, Egypt

**Keywords:** Binary secretary bird optimization algorithm, Secretary bird optimization algorithm (SBOA), Metaheuristic optimization, Feature selection, Evolutionary algorithms, Machine learning, Computational biology and bioinformatics, Engineering, Mathematics and computing

## Abstract

Feature selection is a key step in machine learning–based decision systems, especially in medical and biomedical applications, where datasets often contain a large number of features that can negatively affect both accuracy and interpretability. In this study, we introduce the binary secretary bird optimization algorithm (B-SBOA), a binary version of the secretary bird optimization algorithm specifically developed for feature selection tasks. The proposed approach translates the hunting and escape behaviors of the secretary bird into effective binary search strategies, allowing a well-balanced trade−off between exploration and exploitation. B-SBOA was tested on twenty-five benchmark datasets from the UCI repository and compared with nine well-known binary metaheuristic algorithms, including PSO, GWO, MPA, HBO, SMA, SFOA, DOA, SCA, and MSO. The experimental results show that B-SBOA consistently delivers performance that is either superior to or competitive with existing methods across F-score, precision, recall, and other standard metrics. B-SBOA was evaluated on 25 benchmark datasets from the UCI repository and compared with nine well-known binary metaheuristic algorithms. The results show that B-SBOA achieves superior performance, with average improvements reaching 3–8% in F-score and 2–6% in precision and recall compared to competing methods. In several high-dimensional datasets such as Arrhythmia and Hillvalley, the proposed method achieved the highest classification accuracy while reducing the number of selected features.

## Introduction

In many optimization tasks, metaheuristic algorithms are used because they can handle complex search spaces with limited assumptions. These algorithms usually begin with randomly generated solutions, which helps diversify the search and reduces the risk of settling in local optima. Effective performance is achieved by balancing exploration of the search space with exploitation of promising solutions. Their adaptability makes them suitable for a wide range of applications across different scientific domains^1^.

**Fig. 1:**
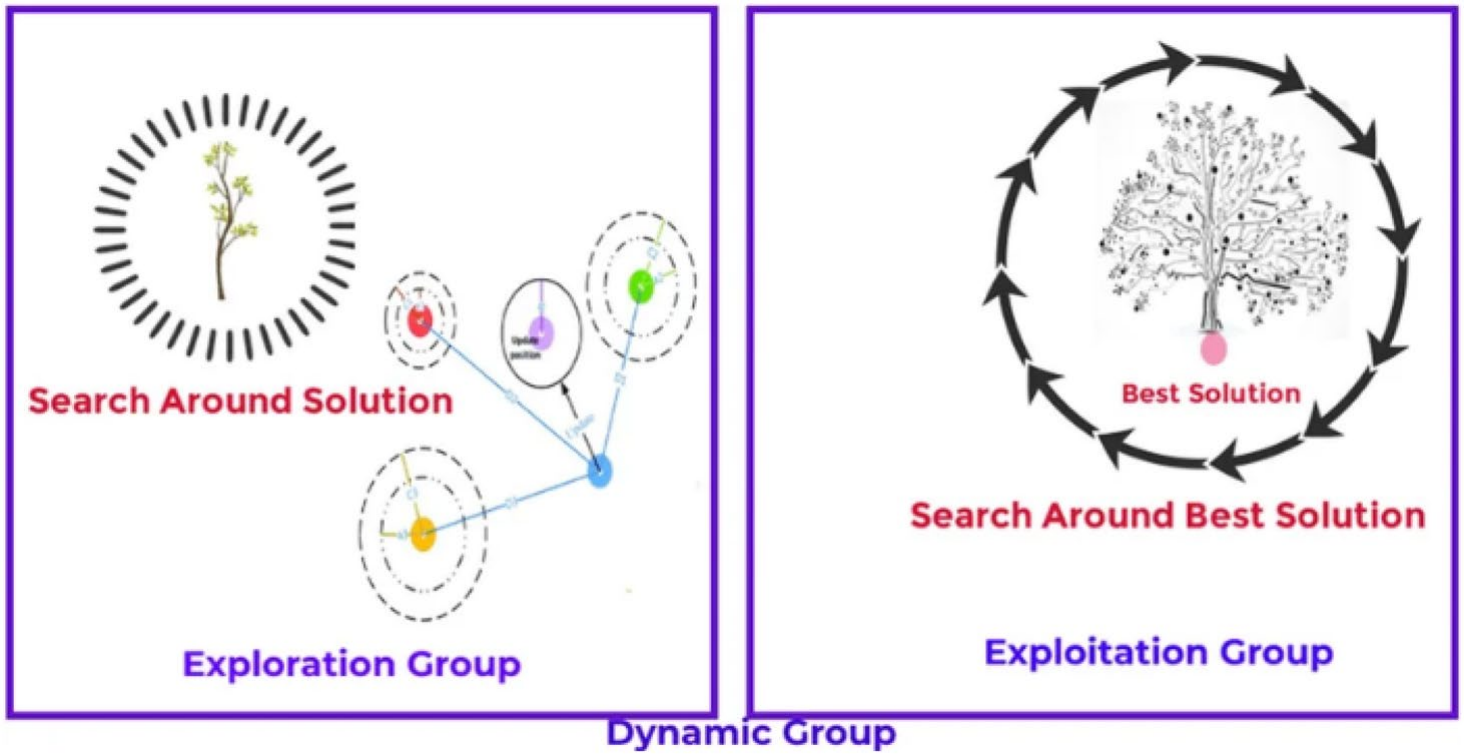
Exploitation vs exploration^3^.

A meta-heuristic aims to enhance efficiency by exploring the search space to find optimal solutions. There are two basic Concepts in metaheuristics as shown in Fig. 1: exploration involves searching the entire feasible region, while exploitation refers to searching the neighborhood of a promising region. One of the most critical aspects of any meta-heuristic strategy is achieving The right balance between exploration and exploitation. While excessive exploration can lead to slow convergence and inefficiency, excessive exploitation may cause premature convergence to a suboptimal solution^2^.

Despite the significant progress that metaheuristic algorithms have made in various fields, they also face some challenges and issues, including susceptibility to getting stuck in local optima, slow convergence rates, insufficient robustness, and high computational costs authors in^4^ explicitly stated that the significant performance of an algorithm in solving a specific set of optimization problems does not guarantee that it will perform equally well in other optimization problems .

Traditional optimization methods and early heuristic approaches have been widely applied to feature selection tasks. However, these methods frequently struggle with large−scale, complex problems, often exhibiting slow convergence, limited search precision, and a tendency to become trapped in local optima. Evolutionary and swarm intelligence algorithms, such as particle swarm optimization (PSO), grey wolf optimizer (GWO), and slime mould algorithm (SMA), have shown promise; however, they still face challenges in achieving a robust balance between exploration and exploitation—particularly in binary feature selection scenarios.

In recent years, optimization approaches inspired by natural processes have gained considerable interest for solving challenging problems. The secretary bird optimization algorithm (SBOA) is one such method that has shown consistent performance, due to its reliable search behavior and steady convergence. Its design allows the algorithm to explore the search space broadly while gradually refining promising solutions.

In this paper, we present a binary version of the SBOA algorithm to address feature selection and optimization problems. The proposed method binarizes the original SBOA, allowing efficient selection for the most relevant features while excluding redundant information. The principal contributions of this study include:

- Introducing the binarization of the secretary bird optimization algorithm. (SBOA) for feature selection tasks.
- Testing and evaluating the proposed binary secretary bird optimization algorithm. (B-SBOA) on 25 benchmark UCI datasets.
- Comparing the performance of B-SBOA with nine state−of-the−art binary optimization algorithms, including SFOA^5^, MSO^6^,DOA^7^, GWO^8^,HBO^9^,MPA^10^,PSO^11^,SMA^12^,SCA^13^.
- Demonstrating that B-SBOA consistently outperforms or matches other algorithms in terms of F-score, precision, and recall, especially for complex high-dimensional datasets.
- Highlighting B-SBOA’s strong global exploration ability, fast convergence, and robustness in both stable and noisy environments.

The remainder of this paper is organized as follows. Section "Related work" reviews previous studies related to feature selection methods. Section "Secretary bird optimization algorithm" introduces the main concepts of the SBOA algorithm and describes the proposed B-SBOA framework. The experimental results are presented and discussed in Sections "Experimental results" and "Discussion". Finally, Section "Conclusion and future directions" concludes the paper and outlines possible directions for future research.

## Related work

**Fig. 2:**
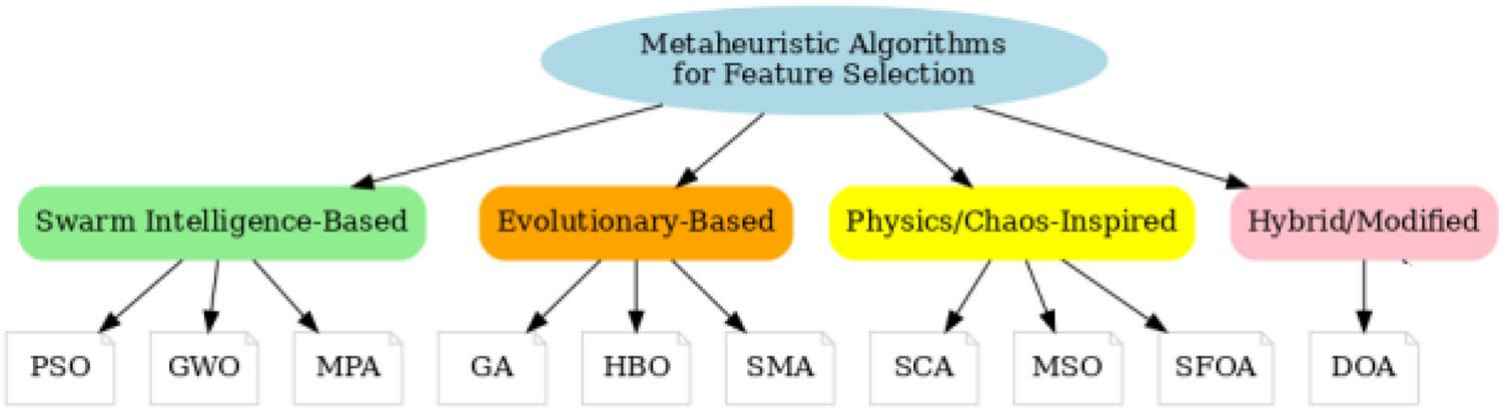
Metaheuristic taxonomy.

Due to the huge amount of data having multidimensional features, the need for selecting related features is increasing. Redundant or irrelevant features can negatively impact prediction accuracy, overfitting, and the training time of models. Metaheuristic approaches provide a flexible way to explore the search space and reduce the risk of getting trapped in poor local solutions. However, as emphasized by the “No Free Lunch Theorem,” no single algorithm performs best across all problems^4^. This section reviews recent metaheuristics for feature selection according to the taxonomy shown in Fig. 2, outlining their key innovations, strengths, and limitations.

Particle swarm optimization (PSO) is a computational optimization technique that is based on the collective behaviour of particles in a swarm. In PSO algorithms, the swarm consists of a set of particles, each of which represents a potential solution to the optimization problem. Each particle in the swarm has a position and a velocity that are updated based on its own experience and the information shared by other particles. In PSO, each particle updates its position by considering both its own best solution and the best solution identified by the swarm. One of the main strengths of PSO lies in its ability to explore large and complex search spaces without requiring gradient information or prior knowledge of the objective function. Moreover, its simple structure makes PSO easy to implement and suitable for a wide range of optimization problems. Despite these advantages, PSO also presents several limitations. For example, the algorithm can be sensitive to the choice of hyperparameters, such as the acceleration coefficients and the inertia weight. Additionally, the algorithm can be computationally intensive, especially for large problems, which can make it challenging to scale to real-world applications^14^.

Grey wolf optimization (GWO) is one of the new meta-heuristic optimization algorithms, which was introduced by Mirjalili et al.^8^. GWO is inspired by social hierarchy and the intelligent hunting method of grey wolves, four types of grey wolves such as alpha, beta, delta, and omega were employed for simulating the leadership hierarchy. Different binary versions was then introduced such as (bGWO1) and (bGWO2) for feature selection problems and the results were effective and efficient in selecting optimal feature subsets for classification problems^15^. However authors in^16^ that it has good performance for the optimization problem whose optimal solution is 0, however, for other problems, its advantage is not as obvious as before or even worse. Also standard GWO can suffer from premature convergence and sensitivity to parameter settings^17^

Marine predators algorithm (MPA) and is inspired by the widespread foraging strategy namely Lévy and Brownian movements in ocean predators along with optimal encounter rate policy in biological interaction between predator and prey. The Marine predators algorithm (MPA) has been widely applied to a range of optimization problems across different research fields. Owing to its simple structure, flexible implementation, and limited number of control parameters, MPA has shown competitive performance in terms of convergence speed and solution accuracy when compared with many existing metaheuristic algorithms. Several improved and modified versions of MPA have been proposed in the literature, as reported in^18^. Some disadvantages of MPA lies behind the division of the MPA optimization process into three phases which make it prone to premature convergence. Besides, all the search space solutions are not optimally explored^19^, and it is unable to generate a diverse initial population with high effectiveness^20^

As one of the earliest evolutionary optimizers, genetic algorithms (GAs) have attracted significant attention due to their effectiveness in addressing complex optimization problems across a wide range of application domains. By employing evolutionary operators such as selection, crossover, and mutation, GAs mimic the principles of natural selection, genetic recombination, and adaptation. This evolutionary process enables them to efficiently explore and exploit complex solution spaces. As a result, the use of GAs has expanded rapidly in numerous fields, including scheduling optimization, the traveling salesman problem (TSP), support vector machines (SVM), pipeline network optimization, vehicle routing problems (VRP), product design, and operations management.^21^ Although genetic algorithms are powerful, they come with a significant drawback: high computational cost. When used for feature selection, GAs typically operate as wrapper methods, which means they must evaluate a large number of candidate feature sets. These repeated evaluations can be very time−consuming, especially for datasets with many instances. As a result, most studies that use GAs for feature selection tend to focus on smaller datasets, usually with fewer than 1000 samples.^22^.

Heap-based optimizer (HBO) utilizes a heap data structure to map the corporate rank hierarchy (CRH) concept. The mathematical model of HBO is built on three pillars: the interaction between the subordinates and their immediate boss, the interaction between the colleagues, and self-contribution of the employees^9^. Several versions were proposd to overcome its deficiencies such as poor search ability and low search efficiency in solving complex optimization problems, In^23^ an improved HBO was presented, based on three new updating strategies (TUS-HBO) and the experimental tests showed that TUS-HBO outperforms HBO. In^24^ binary adaptations of the heap-based optimizer B_HBO are presented and used to determine the optimal features for classifications in wrapping form, The comparison analysis demonstrates that is superior or equivalent to the other algorithms used in the literature.

Slime optimization algorithm (SMA) has recently received much attention from researchers because of its simple structure, excellent optimization capabilities, and acceptable convergence in dealing with various types of complex real world problems. It simulates oscillation and foraging behaviors of slime moulds, the basic version of SMA is difficult to deal with various optimization challenges effectively and comprehensively. A brief summary of the relevant modified SMA variants was presented in^25^.

Sine cosine algorithm (SCA). employs mathematical sine and cosine functions to control the position of solutions, multiple initial random candidate solutions are created and requires them to fluctuate outwards or towards the best solution using a mathematical model based on sine and cosine functions^26^. Despite its popularity, the SCA has limitations in terms of low diversity, stagnation in local optima, and difficulty in achieving global optimization, particularly in complex large−scale problems^26^.

Mirage search optimization (MSO) is a metaheuristic optimization algorithm inspired by physical mirage phenomena and was introduced in^27^. The algorithm operates through two main update mechanisms, referred to as the superior and inferior mirage strategies. These mechanisms support global exploration and local exploitation, respectively, allowing the search process to adapt to different stages of optimization. While MSO shows strong ability to explore the search space, its performance tends to vary across datasets, leading to less consistent results in some cases.

Starfish optimization algorithm (SFOA) mimics behaviors of starfish, including exploration, preying, and regeneration. Its advantage lies in flexibility and exploration, but like other nature−inspired methods, it risks slow convergence in complex feature selection tasks^28^.

The dream optimization algorithm (DOA) is inspired by how human dreams function, where some memories are retained, others are forgotten, and ideas are reorganized in a logical way. These behaviors closely resemble how metaheuristic optimization works. DOA uses a basic memory mechanism along with strategies for forgetting and adding new information to balance exploration and exploitation. It also includes a dream-sharing mechanism that helps the algorithm escape local optima. However, despite these strengths, DOA suffers from high computational complexity, which makes it less suitable for real-time applications.^29^.

A recent study in^30^ introduced the binary golden eagle optimizer with a time−varying flight length (BGEO-TVFL) for feature selection. The method builds on the golden eagle optimizer, originally designed for continuous optimization, and adapts it to binary search spaces via transfer functions. Several transfer functions were examined to identify the most suitable mapping approach. In addition, a time−varying flight-length strategy was used to balance exploration and exploitation during the search process. The method was evaluated on multiple UCI datasets and standard benchmark functions, and the results showed consistent improvements over traditional filter methods and widely used metaheuristic approaches such as PSO, GWO, GA, and ACO. These findings suggest that incorporating adaptive mechanisms can improve the effectiveness of feature selection in high-dimensional problems.

Another recent study^31^ presented a deep learning approach for classifying the severity of diabetic retinopathy using fundus images. The process starts with image preprocessing to enhance contrast and reduce noise, followed by feature extraction using an attention-based transformer model. To improve efficiency and reduce dimensionality, an optimization-based method is then applied to select the most relevant features. The final classification is carried out using a compact pyramidal dense mixed attention network, which is designed to handle multi-class severity levels. The model was evaluated on the APTOS-2019 and DDR datasets, where it achieved high accuracy and showed better performance compared to several existing methods. These results indicate that combining deep learning with optimization techniques can improve the reliability of disease severity prediction.

Table 1 summarizes the related work section as follows:

**Table 1:** Comparison of metaheuristic algorithms for feature selection.

Algorithm	Strengths	Limitations
PSO	Simple, fast convergence	Premature convergence
GWO	Balanced search	Can get stuck in local optima
MPA	Robust global exploration	Computational overhead
GA	Flexible, discrete search	High cost; parameter sensitivity
HBO	Rapid convergence	Scalability issues
SMA	Maintains diversity	High CPU cost
SCA	Simple, global search	Weak local exploitation
MSO	Strong exploration	Unstable across datasets
SFOA	Flexible exploration	Slow convergence
DOA	High diversity	High complexity; limited real-time use

## Secretary bird optimization algorithm

This section first introduces the secretary bird optimization algorithm. Secondly, the binary version is explained.

The secretary bird (Scientifc name: Sagittarius serpentarius) is a striking African raptor known for its distinctive appearance and unique behaviors. The secretary bird is well known for its distinctive and highly effective hunting strategy. Its diet mainly consists of insects, reptiles, small mammals, and other small organisms. When prey is detected, the bird quickly approaches and captures it using its strong legs and sharp talons. The prey is then repeatedly struck against the ground until it is muted.

One of the most distinctive abilities of the secretary bird is its effectiveness in hunting snakes. The bird uses its height and sharp vision to observe the snake’s movements carefully. Through experience gained from repeated encounters, it can anticipate the snake’s actions and remain in control during the hunt. By moving around the snake and provoking reactions while avoiding attacks, the secretary bird continues until the prey is completely hushed^32^.

### Mathematical modeling of the secretary bird optimization algorithm (SBOA)

The secretary bird optimization algorithm (SBOA) is a population-based metaheuristic. Each individual bird in the population represents a candidate solution in the search space, with its position encoding the decision variables of the optimization problem.

#### Initial preparation phase

The initial population is randomly distributed within the lower and upper bounds of the problem:

1 $$\begin{aligned} X_{i,j} = lb_j + r \times (ub_j - lb_j), \quad i = 1,2,\dots ,N; \; j = 1,2,\dots ,Dim \end{aligned}$$

where $X_{i,j}$ is the $j^{th}$ dimension of the $i^{th}$ bird, $lb_j$ and $ub_j$ are bounds, and $r \in [0,1]$ is a random scalar.

The population matrix is represented as:

2 $$\begin{aligned} X = \begin{bmatrix} x_{1,1} & x_{1,2} & \cdots & x_{1,Dim} \\ x_{2,1} & x_{2,2} & \cdots & x_{2,Dim} \\ \vdots & \vdots & \ddots & \vdots \\ x_{N,1} & x_{N,2} & \cdots & x_{N,Dim} \end{bmatrix}_{N \times Dim} \end{aligned}$$

The fitness values of all candidate solutions are computed as:

3 $$\begin{aligned} F = [f(X_1), f(X_2), \dots , f(X_N)] \end{aligned}$$

### Exploration phase

The exploration stage simulates hunting behavior and is divided into three stages.

#### Stage 1: searching for prey

Birds move towards potential prey using Brownian motion:

4 $$\begin{aligned} X^{new}_{i,j} = x_{best} + RB \times (x_{best} - x_{i,j}), \quad 0< t < \tfrac{1}{3}T \end{aligned}$$

where $RB = randn(1,Dim)$ is a Gaussian-distributed vector. The greedy selection rule is:

5 $$\begin{aligned} X_i = {\left\{ \begin{array}{ll} X^{new}_{i}, & f(X^{new}_i) < f(X_i) \\ X_i, & \text {otherwise} \end{array}\right. } \end{aligned}$$

#### Stage 2: consuming prey

The position update is given by:

6 $$\begin{aligned} RB&= randn(1,Dim) \end{aligned}$$

7 $$\begin{aligned} X^{new,P1}_{i,j}&= x_{best} + \exp \left( \left( \frac{t}{T}\right) ^4 \right) \times (RB - 0.5) \times (x_{best} - x_{i,j}), \quad \tfrac{1}{3}T< t < \tfrac{2}{3}T \end{aligned}$$

Greedy selection applies as:

8 $$\begin{aligned} X_i = {\left\{ \begin{array}{ll} X^{new,P1}_i, & f(X^{new,P1}_i) < f(X_i) \\ X_i, & \text {otherwise} \end{array}\right. } \end{aligned}$$

#### Stage 3: attacking prey

Levy flight is introduced:

9 $$\begin{aligned} X_i = {\left\{ \begin{array}{ll} X^{new,P1}_i, & f(X^{new,P1}_i) < f(X_i) \\ X_i, & \text {otherwise} \end{array}\right. } \end{aligned}$$

Levy step length is defined as:

10 $$\begin{aligned} RL= & 0.5 \times Levy(Dim) \end{aligned}$$

11 $$\begin{aligned} Levy(D)= & \frac{s \times u}{|v|^{1/\beta }}, \quad u,v \sim N(0,\sigma ^2) \end{aligned}$$

12 $$\begin{aligned} \sigma= & \left( \frac{ \Gamma (1+\beta ) \times \sin (\pi \beta /2)}{\Gamma ((1+\beta )/2) \times \beta \times 2^{(\beta -1)/2}} \right) ^{1/\beta } \end{aligned}$$

with $$\beta = 1.5$$ typically.

### Exploitation phase

Escaping predator behavior is modeled using two strategies:

13 $$\begin{aligned} X^{new,P2}_{i,j} = {\left\{ \begin{array}{ll} C1: \; x_{best} + (2RB-1) \times \left( 1-\frac{t}{T}\right) ^2 \times x_{i,j}, & r < r_i \\ C2: \; x_{i,j} + R2 \times (x_{random} - K \times x_{i,j}), & \text {otherwise} \end{array}\right. } \end{aligned}$$

with greedy selection:

14 $$\begin{aligned} X_i = {\left\{ \begin{array}{ll} X^{new,P2}_i, & f(X^{new,P2}_i) < f(X_i) \\ X_i, & \text {otherwise} \end{array}\right. } \end{aligned}$$

where *K* is defined as:

15 $$\begin{aligned} K = round(1 + rand(1,1)) \end{aligned}$$

### Binary secretary bird optimization algorithm (B-SBOA)

#### Representation and initialization

Each candidate solution is represented as a binary string $X_i \in \{0,1\}^{dim}$. The initial population is created by thresholding random numbers from an RNG *rndGen*:

16 $$\begin{aligned} X_{i,j} = {\left\{ \begin{array}{ll} 1, & \text {if } rndGen(1,1) > 0.5, \\ \\ 0, & \text {otherwise}. \end{array}\right. } \end{aligned}$$

All candidates are evaluated and the best solution $Best\_pos$ with fitness *best* is stored.

***Stages.*** B-SBOA simulates three hunting stages and an escape strategy:

- **Stage 1 (Exploration,** $t<T/3$**):** Flip bits guided by the XOR of two random peers. The update rule is
  17 $$\begin{aligned} . X_i^{(t+1)} = X_i^{(t)} \oplus \Big ( (X_{p}^{(t)} \oplus X_{q}^{(t)}) \wedge M \Big ), \end{aligned}$$
  where $X_p, X_q$ are two random peers, $M \sim \textrm{Bernoulli}(\mu )^{Dim}$, $\oplus$ is XOR, and $\wedge$ is AND.
- **Stage 2 (Approach,** $T/3\le t<2T/3$**):** Move toward the best solution by flipping a subset of differing bits:
  18 $$\begin{aligned} X_i^{(t+1)} = X_i^{(t)} \oplus \Big ( (X_{best}^{(t)} \oplus X_i^{(t)}) \wedge M \Big ), \end{aligned}$$
  where $$M \sim \textrm{Bernoulli}(\mu )^{Dim}$$.
- **Stage 3 (Attack,** $t\ge 2T/3$**):** Intensified local search by flipping bits with contraction factor probability:
  19 $$\begin{aligned} X_i^{(t+1)} = X_i^{(t)} \oplus J, \end{aligned}$$
  where $$J \sim \textrm{Bernoulli}(CF(t))^{Dim}$$ and
  20 $$\begin{aligned} CF(t) = \Big (1 - \frac{t}{T}\Big )^{\tfrac{2t}{T}}. \end{aligned}$$
- *Escape strategies* Two exploitation strategies are used with equal probability:
  21 $$\begin{aligned} X_i^{(t+1)} = X_{best}^{(t)} \oplus M_1, \quad M_1 \sim \textrm{Bernoulli}\Big ((1 - t/T)^2\Big )^{Dim}, \end{aligned}$$
  which jitters around the best solution (C1), or
  22 $$\begin{aligned} X_i^{(t+1)} = X_i^{(t)} \oplus \Big ( (X_r^{(t)} \oplus X_i^{(t)}) \wedge M_2 \Big ), \quad M_2 \sim \textrm{Bernoulli}(\mu )^{Dim}, \end{aligned}$$
  where $X_r$ is a random peer (C2).

Those equations can be interpreted intuitively as follows. The XOR operation represents the difference between candidate solutions, allowing the algorithm to explore new feature subsets. The Bernoulli mask controls how many bits are flipped, which directly affects the balance between exploration and exploitation. In all cases, a greedy selection mechanism is used, in which a new solution replaces the current one only if it yields an equal or better fitness value.

In early iterations, larger bit changes encourage diversity, while in later iterations smaller updates refine the solution around the best candidate. The contraction factor (CF) begins with a slow reduction in the early iterations to preserve exploration and accelerates in later iterations to strengthen exploitation.

The pseudo-code is given below:

**Algorithm 1:**
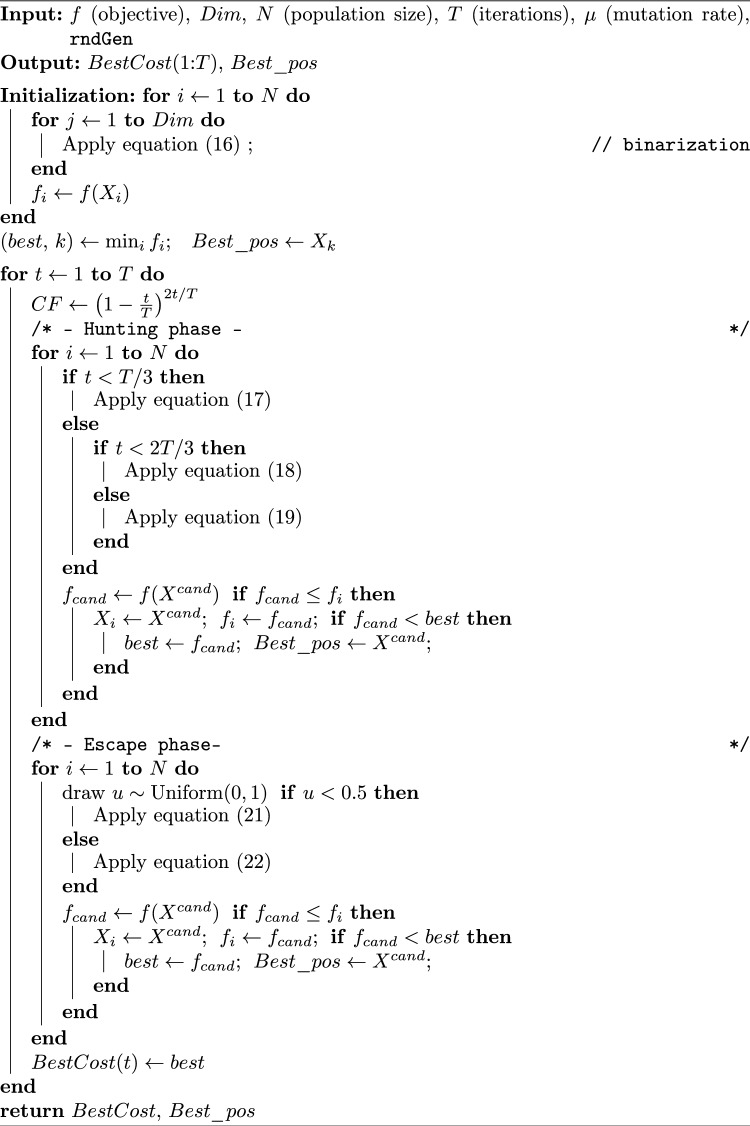
Binary secretary bird optimization algorithm (B-SBOA).

### Complexity analysis

The proposed Binary secretary bird optimization algorithm (B-SBOA) is analyzed in terms of time and space complexity.

#### Time complexity

The time complexity of the B-SBOA is influenced by the number of candidate solutions (population size, $N$) and the number of iterations ($T$).

In each iteration, the algorithm updates the position of each bird (candidate solution) based on the current exploration and exploitation phases. For each bird, this update involves calculations such as XOR operations, fitness evaluations, and random number generation. Hence, the overall time complexity is $O(N \times T)$.

However, the complexity of evaluating the fitness of each solution depends on the specific problem being solved. If the feature selection problem involves high-dimensional data, the complexity of evaluating each candidate increases based on the number of features, $D$. Thus, the time complexity of the B-SBOA becomes $O(N \times T \times D)$, where $D$ is the dimensionality of the dataset.

#### Space complexity

The space complexity of the B-SBOA arises from storing the positions of the birds (solutions) and their corresponding fitness values.

Each bird’s position is represented as a binary string of length $D$, meaning that each bird requires $D$ bits of memory. Therefore, for $N$ birds, the space complexity for storing the population is $O(N \times D)$.

Additionally, for each iteration, the algorithm stores temporary variables for each bird (e.g., updated positions and fitness values). As a result, the overall complexity of the space is $O(N \times D)$.

#### Convergence behavior

The B-SBOA exhibits rapid convergence, as demonstrated in the experimental results. The balance of exploration-exploitation of the algorithm enables quick convergence to near-optimal solutions. The convergence rate is efficient, especially for high-dimensional datasets, suggesting that the algorithm significantly reduces the classification error within the first few hundred evaluations.

#### Exploration vs. exploitation

The algorithm balances exploration and exploitation to prevent premature convergence. During the exploration phase, the algorithm performs a broad search in the feature space. In the exploitation phase, the algorithm refines its search to focus on the most promising areas. This balance improves algorithm performance by avoiding stagnation in local optima and accelerating convergence to optimal solutions.

### Conclusion

In summary, the time complexity of the B-SBOA is $O(N \times T \times D)$, where $N$ is the population size, $T$ is the number of iterations, and $D$ is the number of features. The algorithm is efficient and performs well on high-dimensional data, delivering optimal solutions with fast convergence while maintaining a good balance between exploration and exploitation.

## Experimental results

To evaluate the performance of the proposed SBOA algorithm, a set of experiments were conducted to examine its effectiveness and limitations. Feature selection was performed on 25 datasets with varying dimensionality, including both low- and high-dimensional cases. The results obtained by the proposed method were compared with several established population-based metaheuristic algorithms.

**Fig. 3:**
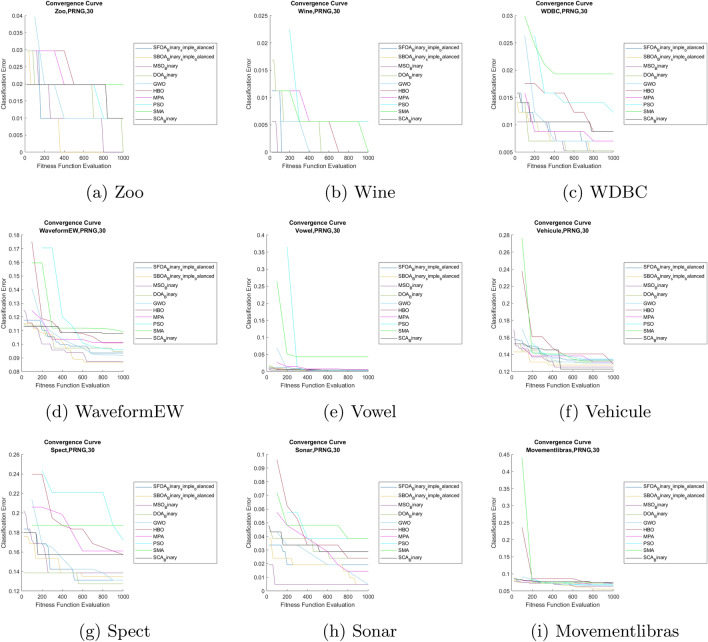
Convergence curves across datasets (Part 1).

**Fig. 4:**
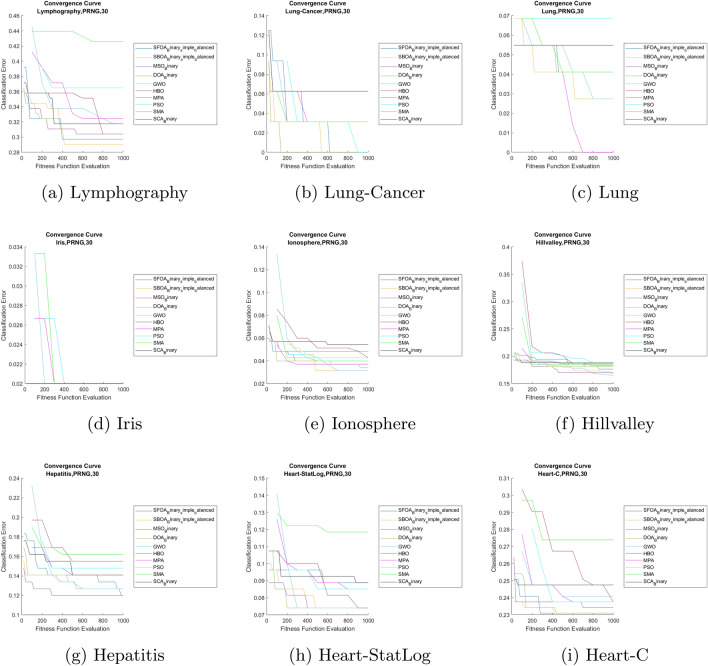
Convergence curves across datasets (Part 2).

**Fig. 5:**
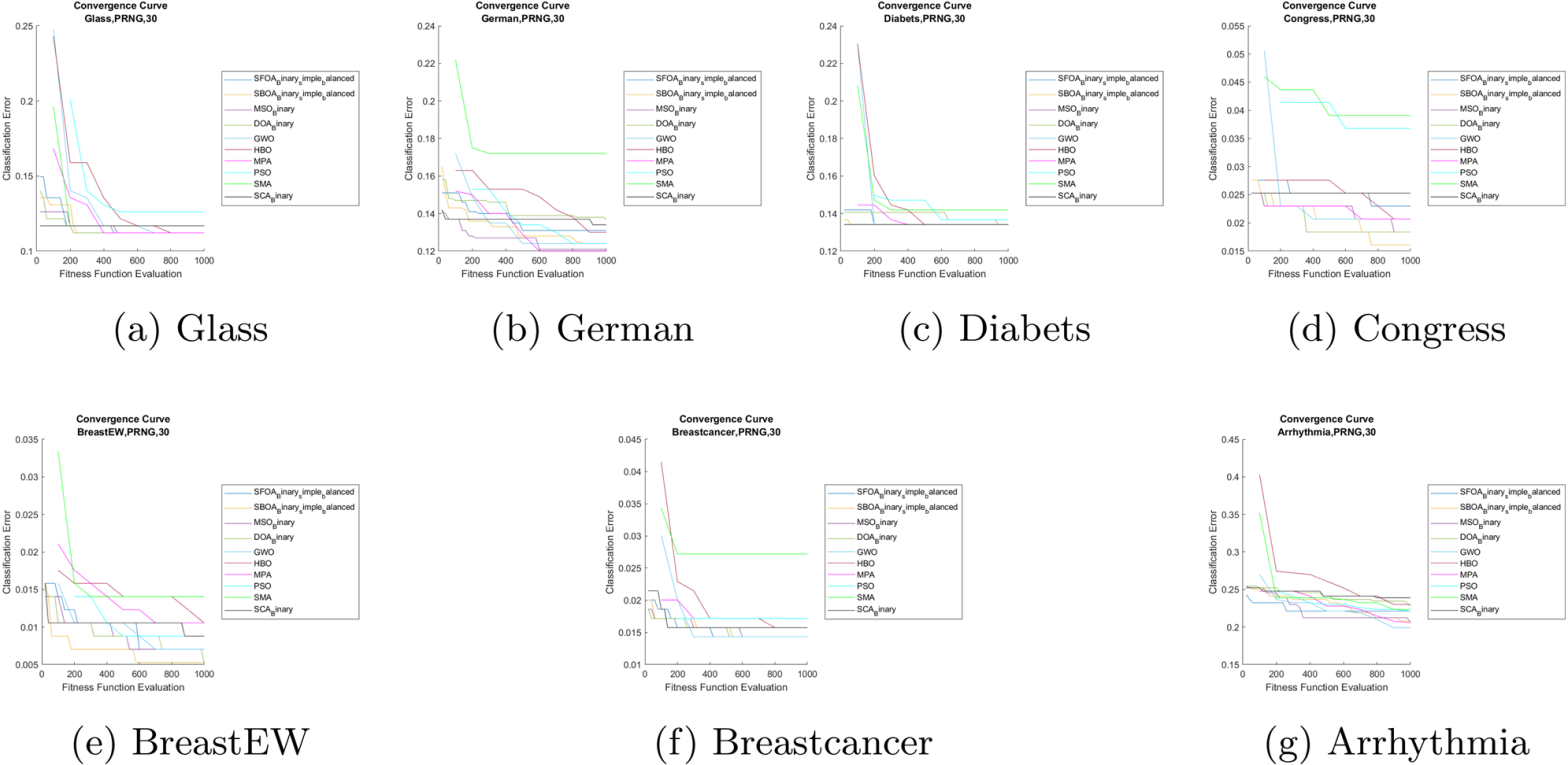
Convergence curves across datasets (Part 3).

We utilize various of datasets. Most of these datasets were obtained from the UCI machine learning repository (Figs. 3, 4, 5). A detailed description of the datasets is presented in Table 2.

**Table 2:** Data sets description.

Data set	Features number	Instance number
Arrhythmia	278	452
Hepatitis	19	142
Breast Cancer	10	699
BreastEW	30	569
Congress	435	16
Diabetes	8	768
German	24	1000
Glass	9	214
Heart-C	13	303
Heart-StatLog	13	270
Vehicule	18	846
Hillvalley	100	606
Ionosphere	34	351
Iris	4	150
Lung	73	325
Lung-Cancer	56	32
Lymphography	18	148
Movementlibras	90	360
Sonar	60	208
Spect	22	267
Zoo	16	101
Vowel	901	10
WaveformEW	40	5000
WDBC	31	569
Wine	13	178

### Convergence curve

Across all 25 benchmark datasets, the binary secretary bird optimization algorithm (B-SBOA) exhibits consistently faster and smoother convergence toward minimal classification error compared to competing algorithms. In most datasets, B-SBOA (and its balanced variant) rapidly descends to a near-optimal solution within the first 200–400 evaluations, while maintaining stability in later iterations. This pattern indicates an efficient balance between exploration (diverse search early on) and exploitation (precise fine−tuning in later stages).

### Measurements

The results of the feature selection process are analyzed using several evaluation metrics, including F-score ,max, mean, min, precision , recall, and number of selected features.

Tables 3, 4, 5,6, 7 and 8 illustrates the results of the proposed Binary SBOA compared with other algorithms for feature selection on various datasets.

**Table 3:** Fscore across all datasets (higher is better).

Dataset	SBOA	SFOA	MSO	DOA	GWO	HBO	MPA	PSO	SMA	SCA
Arrhythmia	**0.619**	0.562	0.594	0.579	0.594	0.562	0.572	0.571	0.528	0.549
Breastcancer	**0.984**	**0.984**	**0.984**	0.983	**0.984**	**0.984**	0.982	0.983	0.977	0.983
BreastEW	**0.994**	0.992	0.993	0.992	0.992	0.991	0.991	0.990	0.984	0.990
Congress	**0.981**	0.979	**0.981**	**0.981**	0.979	0.979	0.979	0.974	0.966	0.978
Diabets	**0.851**	**0.851**	**0.851**	**0.851**	0.850	**0.851**	0.850	0.849	0.838	**0.851**
German	**0.851**	0.843	0.850	0.845	0.846	0.838	0.838	0.840	0.794	0.834
Glass	**0.872**	0.869	0.870	**0.872**	0.869	0.870	0.868	0.867	0.841	0.867
Heart-C	**0.654**	0.640	0.651	0.649	0.637	0.636	0.621	0.637	0.598	0.645
Heart-StatLog	**0.924**	0.919	0.922	0.921	0.922	0.920	0.916	0.908	0.890	0.914
Hepatitis	**0.881**	0.869	0.876	0.871	0.869	0.860	0.866	0.859	0.845	0.860
Hillvalley	**0.838**	0.819	0.827	0.822	0.830	0.814	0.824	0.815	0.823	0.812
Ionosphere	**0.965**	0.955	0.961	0.957	0.961	0.952	0.958	0.949	0.953	0.949
Iris	**0.980**	**0.980**	**0.980**	**0.980**	**0.980**	**0.980**	**0.980**	0.978	**0.980**	**0.980**
Lung-Cancer	**0.996**	0.972	0.989	0.983	0.968	0.968	0.978	0.969	0.949	0.959
Lung	**0.970**	0.955	0.963	0.956	0.956	0.956	0.967	0.954	0.961	0.952
Lymphography	0.535	0.519	0.530	0.523	**0.538**	0.516	0.511	0.506	0.447	0.513
Movementlibras	**0.943**	0.934	0.937	0.935	0.937	0.932	0.935	0.933	0.933	0.930
Sonar	**0.994**	0.983	0.986	0.980	0.985	0.974	0.977	0.974	0.955	0.971
Spect	**0.867**	0.853	0.863	0.858	0.856	0.849	0.848	0.843	0.795	0.839
Vehicule	0.875	0.871	**0.876**	0.872	0.871	0.869	0.869	0.867	0.854	0.865
Vowel	**0.906**	**0.906**	**0.906**	**0.906**	**0.906**	**0.906**	**0.906**	0.905	0.896	**0.906**
WaveformEW	**0.914**	0.906	**0.914**	0.907	0.911	0.903	0.901	0.904	0.869	0.896
WDBC	**0.994**	0.992	**0.994**	0.993	0.992	0.991	0.990	0.990	0.986	0.990
Wine	**1.000**	**1.000**	**1.000**	**1.000**	0.999	**1.000**	0.999	0.996	0.994	0.999
Zoo	**0.995**	0.982	0.993	0.988	0.982	0.987	0.980	0.977	0.953	0.980

Significant values are in bold.

The comparison of F-scores across all datasets shows that B-SBOA consistently achieves the best or near-best classification performance among the evaluated algorithms. This strong performance appears in both simple and complex datasets, indicating an effective balance between exploration and exploitation. These results confirm the ability of B-SBOA to select informative feature subsets while maintaining high accuracy and stable convergence (Table 4).

**Table 4:** Precision across all datasets (higher is better).

Dataset	SBOA	SFOA	MSO	DOA	GWO	HBO	MPA	PSO	SMA	SCA
Arrhythmia	**0.749**	0.691	0.729	0.722	0.723	0.701	0.691	0.714	0.608	0.686
Breastcancer	0.981	0.981	0.981	0.981	0.981	0.981	0.980	0.980	0.975	**0.981**
BreastEW	**0.995**	0.994	0.994	0.994	0.993	0.992	0.992	0.992	0.986	0.992
Congress	0.980	0.978	0.980	0.980	0.978	0.978	0.978	0.972	0.964	**0.977**
Diabets	**0.856**	**0.856**	**0.856**	**0.856**	0.855	0.855	0.855	0.854	0.842	0.855
German	**0.870**	0.863	0.872	0.866	0.865	0.858	0.858	0.860	0.813	0.853
Glass	**0.898**	0.889	0.891	0.895	0.887	0.890	0.877	0.881	0.853	0.880
Heart-C	**0.760**	0.733	0.758	0.754	0.731	0.732	0.706	0.739	0.700	0.750
Heart-StatLog	**0.926**	0.920	0.924	0.923	0.923	0.921	0.918	0.910	0.891	0.916
Hepatitis	**0.885**	0.873	0.880	0.875	0.873	0.864	0.870	0.864	0.848	0.865
Hillvalley	**0.839**	0.820	0.829	0.823	0.831	0.815	0.825	0.816	0.823	0.813
Ionosphere	**0.973**	0.967	0.971	0.968	0.971	0.964	0.968	0.962	0.961	0.961
Iris	**0.980**	**0.980**	**0.980**	**0.980**	**0.980**	**0.980**	**0.980**	0.978	**0.980**	**0.980**
Lung-cancer	**0.998**	0.985	0.993	0.991	0.982	0.979	0.988	0.983	0.966	0.974
Lung	0.966	0.949	0.958	0.949	0.951	0.950	**0.961**	0.947	0.958	0.945
Lymphography	0.575	0.555	0.569	0.557	**0.587**	0.553	0.546	0.546	0.497	0.552
Movementlibras	**0.945**	0.937	0.939	0.938	0.939	0.934	0.937	0.936	0.935	0.933
Sonar	**0.994**	0.984	0.986	0.981	0.985	0.975	0.978	0.975	0.956	0.972
Spect	**0.871**	0.859	0.868	0.863	0.860	0.855	0.852	0.850	0.802	0.843
Vehicule	**0.874**	0.869	0.875	0.871	0.870	0.867	0.868	0.865	0.853	0.863
Vowel	**0.906**	**0.906**	**0.906**	**0.906**	**0.906**	**0.906**	0.905	0.905	0.896	0.905
WaveformEW	**0.914**	0.906	**0.914**	0.907	0.911	0.903	0.901	0.904	0.869	0.896
WDBC	**0.996**	0.994	0.995	0.994	0.994	0.993	0.992	0.992	0.988	0.992
Wine	**1.000**	**1.000**	**1.000**	**1.000**	0.999	**1.000**	0.999	0.996	0.993	0.999
Zoo	**0.995**	0.984	0.992	0.988	0.983	0.989	0.982	0.980	0.962	0.981

Significant values are in bold.

The precision results show that SBOA is effective in selecting relevant features while limiting false positive predictions. The algorithm performs consistently well across most datasets, with particularly strong results on noisy and high-dimensional data such as Arrhythmia, BreastEW, and Hepatitis which indicates that SBOA preserves useful feature information, leading to improved classification accuracy (Table 5).

**Table 5:** Recall across all datasets (higher is better).

Dataset	SBOA	SFOA	MSO	DOA	GWO	HBO	MPA	PSO	SMA	SCA
Arrhythmia	**0.528**	0.474	0.502	0.485	0.505	0.470	0.488	0.477	0.469	0.458
Breastcancer	**0.987**	0.986	**0.987**	0.986	0.986	0.986	0.985	0.985	0.979	0.986
BreastEW	**0.993**	0.990	0.992	0.991	0.990	0.989	0.989	0.988	0.982	0.988
Congress	0.982	0.981	**0.983**	0.982	0.981	0.980	0.980	0.975	0.968	0.979
Diabets	0.846	0.846	0.846	0.846	0.846	**0.847**	0.846	0.845	0.834	**0.847**
German	**0.832**	0.824	0.829	0.825	0.827	0.819	0.818	0.821	0.775	0.816
Glass	0.848	0.851	0.851	0.850	0.851	0.851	**0.861**	0.854	0.830	0.856
Heart-C	**0.574**	0.569	0.570	0.571	0.565	0.563	0.556	0.562	0.523	0.567
Heart-StatLog	**0.921**	0.918	0.920	0.919	0.920	0.918	0.914	0.906	0.889	0.913
Hepatitis	**0.876**	0.865	0.872	0.868	0.865	0.856	0.863	0.854	0.842	0.855
Hillvalley	**0.837**	0.818	0.826	0.821	0.829	0.813	0.823	0.814	0.822	0.811
Ionosphere	**0.956**	0.944	0.952	0.946	0.952	0.940	0.948	0.937	0.944	0.936
Iris	**0.980**	**0.980**	**0.980**	**0.980**	**0.980**	**0.980**	**0.980**	0.978	**0.980**	**0.980**
Lung-Cancer	**0.994**	0.959	0.984	0.976	0.955	0.958	0.969	0.957	0.933	0.945
Lung	**0.975**	0.962	0.968	0.963	0.962	0.962	0.972	0.961	0.964	0.960
Lymphography	**0.502**	0.489	0.498	0.495	0.497	0.486	0.481	0.474	0.410	0.480
Movementlibras	**0.941**	0.932	0.935	0.933	0.935	0.929	0.932	0.931	0.930	0.927
Sonar	**0.993**	0.982	0.985	0.979	0.984	0.973	0.976	0.974	0.954	0.969
Spect	**0.863**	0.847	0.858	0.853	0.853	0.843	0.843	0.836	0.789	0.835
Vehicule	**0.877**	0.872	**0.877**	0.873	0.872	0.870	0.870	0.868	0.855	0.866
Vowel	**0.907**	**0.907**	**0.907**	**0.907**	**0.907**	**0.907**	0.906	0.906	0.897	0.906
WaveformEW	**0.914**	0.906	**0.914**	0.907	0.911	0.903	0.901	0.904	0.869	0.896
WDBC	**0.993**	0.991	0.992	0.991	0.990	0.989	0.989	0.989	0.983	0.987
Wine	**1.000**	**1.000**	**1.000**	**1.000**	**1.000**	**1.000**	0.999	0.997	0.995	**1.000**
Zoo	**0.995**	0.981	0.993	0.989	0.980	0.986	0.978	0.974	0.943	0.978

Significant values are in bold.

The recall results show that SBOA is effective in selecting relevant instances in almost all datasets, which means that important cases are rarely missed. Although methods such as GWO and MSO achieve similar recall in some datasets, their performance is less consistent. SBOA, on the contrary, provides more stable recall results across different datasets. This suggests that the algorithm does not remove useful features too early and maintains a good balance between precision and recall (Table 6).

**Table 6:** Max values across all datasets (lower is better).

Dataset	SBOA	SFOA	MSO	DOA	GWO	HBO	MPA	PSO	SMA	SCA
Arrhythmia	**0.208**	0.237	0.226	0.235	0.223	0.243	0.237	0.250	0.252	0.248
Breastcancer	**0.016**	**0.016**	**0.016**	0.017	0.017	0.017	0.017	0.019	0.031	0.017
BreastEW	**0.009**	0.009	0.009	0.011	0.012	0.011	0.012	0.012	0.021	0.012
Congress	**0.021**	0.023	**0.021**	**0.021**	0.023	0.023	0.025	0.041	0.044	0.025
Diabets	**0.134**	**0.134**	**0.134**	**0.134**	0.145	**0.134**	0.145	0.145	0.177	**0.134**
German	**0.132**	0.135	0.129	0.137	0.138	0.141	0.143	0.141	0.223	0.140
Glass	**0.121**	**0.121**	0.117	**0.121**	0.131	0.126	0.131	0.150	0.164	0.117
Heart-C	**0.238**	0.241	0.241	0.241	0.248	0.244	0.248	0.257	0.304	0.248
Heart-StatLog	**0.085**	0.089	0.081	0.085	0.085	0.085	0.089	0.133	0.159	0.089
Hepatitis	**0.134**	0.148	0.141	0.141	0.141	0.155	0.155	0.162	0.176	0.155
Hillvalley	**0.173**	0.188	0.183	0.188	0.186	0.195	0.193	0.193	0.193	0.195
Ionosphere	**0.040**	0.048	0.043	0.046	0.046	0.054	0.046	0.060	0.054	0.054
Iris	**0.020**	**0.020**	**0.020**	**0.020**	**0.020**	**0.020**	**0.020**	0.033	0.033	**0.020**
Lung-Cancer	**0.031**	**0.031**	**0.031**	**0.031**	0.062	0.062	**0.031**	0.062	0.062	0.062
Lung	**0.055**	**0.055**	**0.055**	**0.055**	**0.055**	**0.055**	**0.055**	0.068	**0.055**	**0.055**
Lymphography	**0.311**	0.324	0.318	0.324	0.345	0.345	0.345	0.365	0.426	0.345
Movementlibras	**0.064**	0.075	0.069	0.072	0.075	0.078	0.075	0.081	0.081	0.078
Sonar	**0.014**	0.029	0.024	0.034	0.029	0.034	0.034	0.038	0.077	0.038
Spect	**0.142**	0.154	**0.142**	0.146	0.154	0.161	0.161	0.225	0.232	0.169
Vehicule	**0.130**	0.138	**0.130**	0.135	0.138	0.139	0.142	0.144	0.173	0.143
Vowel	**0.004**	**0.004**	**0.004**	**0.004**	0.007	**0.004**	0.006	0.009	0.043	0.006
WaveformEW	**0.092**	0.101	**0.091**	0.100	0.095	0.105	0.107	0.105	0.157	0.109
WDBC	**0.007**	0.011	**0.007**	0.009	0.011	0.011	0.012	0.014	0.021	0.012
Wine	**0.000**	0.006	**0.000**	0.006	0.006	**0.000**	0.006	0.011	0.022	0.006
Zoo	**0.010**	0.020	**0.010**	**0.010**	0.020	0.020	0.020	0.030	0.050	0.020

Significant values are in bold.

Results based on the Max metric indicate that SBOA produces the lowest maximum error, suggesting stable convergence and reduced sensitivity to initialization (Table 7).

**Table 7:** Median values across all datasets (lower is better).

Dataset	SBOA	SFOA	MSO	DOA	GWO	HBO	MPA	PSO	SMA	SCA
Arrhythmia	**0.197**	0.228	0.213	0.226	0.210	0.235	0.219	0.235	0.230	0.241
Breastcancer	**0.014**	0.014	0.014	0.015	0.014	0.014	0.016	0.016	0.020	0.016
BreastEW	**0.005**	0.007	0.007	0.007	0.007	0.009	0.009	0.009	0.016	0.010
Congress	**0.018**	0.020	**0.018**	**0.018**	0.021	0.021	0.021	0.023	0.032	0.021
Diabets	**0.134**	**0.134**	**0.134**	**0.134**	**0.134**	**0.134**	**0.134**	**0.134**	0.145	**0.134**
German	**0.123**	0.129	0.122	0.127	0.124	0.132	0.132	0.131	0.166	0.135
Glass	**0.112**	**0.112**	**0.112**	**0.112**	**0.112**	**0.112**	0.117	0.117	0.136	**0.112**
Heart-C	**0.231**	0.233	**0.231**	**0.231**	0.234	0.234	0.238	0.238	0.267	0.234
Heart-StatLog	**0.074**	0.081	**0.074**	**0.074**	**0.074**	0.080	0.085	0.085	0.107	0.085
Hepatitis	**0.120**	0.130	**0.120**	0.127	0.127	0.141	0.127	0.141	0.155	0.141
Hillvalley	**0.162**	0.182	0.173	0.180	0.170	0.187	0.177	0.188	0.178	0.190
Ionosphere	**0.034**	0.043	0.037	0.040	0.037	0.046	0.040	0.046	0.043	0.048
Iris	**0.020**	**0.020**	**0.020**	**0.020**	**0.020**	**0.020**	**0.020**	**0.020**	**0.020**	**0.020**
Lung-Cancer	**0.000**	0.031	**0.000**	**0.000**	0.031	0.031	0.031	0.031	0.031	0.031
Lung	**0.027**	0.041	0.041	0.041	0.041	0.041	0.041	0.055	0.041	0.055
Lymphography	0.291	0.311	0.301	0.304	0.304	0.318	0.318	0.321	0.385	**0.324**
Movementlibras	**0.058**	0.069	0.065	0.067	0.064	0.071	0.067	0.069	0.069	0.072
Sonar	**0.005**	0.014	0.014	0.019	0.014	0.024	0.024	0.024	0.043	0.029
Spect	**0.131**	0.142	**0.131**	0.139	0.139	0.146	0.146	0.146	0.199	0.157
Vehicule	**0.124**	0.131	**0.124**	0.129	0.128	0.132	0.132	0.134	0.144	0.136
Vowel	**0.003**	0.004	**0.003**	**0.003**	**0.003**	**0.003**	0.004	0.004	0.012	0.004
WaveformEW	**0.087**	0.094	0.086	0.093	0.088	0.098	0.099	0.097	0.131	0.104
WDBC	**0.005**	0.007	0.005	0.007	0.007	0.009	0.009	0.009	0.014	0.010
Wine	**0.000**	**0.000**	**0.000**	**0.000**	**0.000**	**0.000**	**0.000**	0.006	0.006	**0.000**
Zoo	**0.000**	0.010	**0.000**	0.010	0.010	0.010	0.010	0.010	0.020	0.010

Significant values are in bold.

Median results show that SBOA achieves low and consistent classification errors across most datasets, indicating reliable performance.

**Table 8:** Min values across all datasets (lower is better).

Dataset	SBOA	SFOA	MSO	DOA	GWO	HBO	MPA	PSO	SMA	SCA
Arrhythmia	**0.186**	0.215	0.197	0.219	0.186	0.221	0.195	0.208	0.204	0.232
Breastcancer	**0.014**	**0.014**	**0.014**	**0.014**	**0.014**	**0.014**	**0.014**	**0.014**	0.016	**0.014**
BreastEW	**0.004**	0.005	0.005	0.005	0.005	0.007	0.005	0.005	0.007	0.007
Congress	**0.016**	**0.016**	**0.016**	**0.016**	**0.016**	**0.016**	**0.016**	**0.016**	0.021	**0.018**
Diabets	**0.134**	**0.134**	**0.134**	**0.134**	**0.134**	**0.134**	**0.134**	**0.134**	0.134	**0.134**
German	**0.113**	0.114	0.113	0.115	0.113	0.122	0.119	0.114	0.135	0.130
Glass	**0.112**	**0.112**	**0.112**	**0.112**	**0.112**	**0.112**	**0.112**	**0.112**	0.117	**0.112**
Heart-C	**0.228**	**0.228**	**0.228**	**0.228**	**0.228**	**0.228**	**0.228**	0.231	0.244	0.231
Heart-StatLog	**0.074**	**0.074**	**0.074**	**0.074**	**0.074**	**0.074**	**0.074**	**0.074**	0.085	**0.074**
Hepatitis	**0.106**	0.113	**0.106**	0.113	**0.106**	0.120	0.113	0.106	0.120	0.120
Hillvalley	0.155	0.177	0.162	0.170	**0.157**	0.175	0.165	0.175	0.165	0.178
Ionosphere	**0.026**	0.031	0.028	0.034	0.028	0.034	0.028	0.040	0.034	0.037
Iris	**0.020**	**0.020**	**0.020**	**0.020**	**0.020**	**0.020**	**0.020**	**0.020**	**0.020**	**0.020**
Lung-Cancer	**0.000**	**0.000**	**0.000**	**0.000**	**0.000**	**0.000**	**0.000**	**0.000**	**0.000**	**0.000**
Lung	0.014	0.041	0.027	0.027	0.027	0.041	**0.000**	0.027	0.014	0.041
Lymphography	0.291	0.291	0.291	0.291	0.291	0.297	0.297	0.291	0.318	**0.291**
Movementlibras	0.056	0.064	0.061	0.061	**0.053**	0.064	0.058	0.061	0.058	0.064
Sonar	**0.000**	0.010	0.005	0.010	**0.000**	0.019	0.010	0.010	0.029	0.014
Spect	0.116	**0.116**	0.124	0.127	0.124	0.131	0.131	0.127	0.165	0.139
Vehicule	**0.122**	0.119	0.119	0.119	0.122	0.122	0.119	0.119	0.132	0.123
Vowel	**0.003**	0.003	**0.003**	**0.003**	**0.003**	**0.003**	**0.003**	**0.003**	0.006	**0.003**
WaveformEW	**0.082**	0.088	**0.082**	0.087	0.084	0.090	0.091	0.089	0.102	0.098
WDBC	**0.004**	**0.004**	0.005	**0.004**	**0.004**	0.007	0.007	0.005	0.005	0.009
Wine	**0.000**	**0.000**	**0.000**	**0.000**	**0.000**	**0.000**	**0.000**	**0.000**	**0.000**	**0.000**
Zoo	**0.000**	**0.000**	**0.000**	**0.000**	**0.000**	**0.000**	**0.000**	**0.000**	0.010	**0.000**

Significant values are in bold.

Min error results show that SBOA consistently achieves the lowest classification error on most datasets, indicating efficient identification of high-quality feature subsets.

Collectively, these results prove that SBOA delivers not only the highest accuracy but also the most stable and repeatable performance across diverse datasets and problem complexities.

### Box plot behavior analysis

**Fig. 6:**
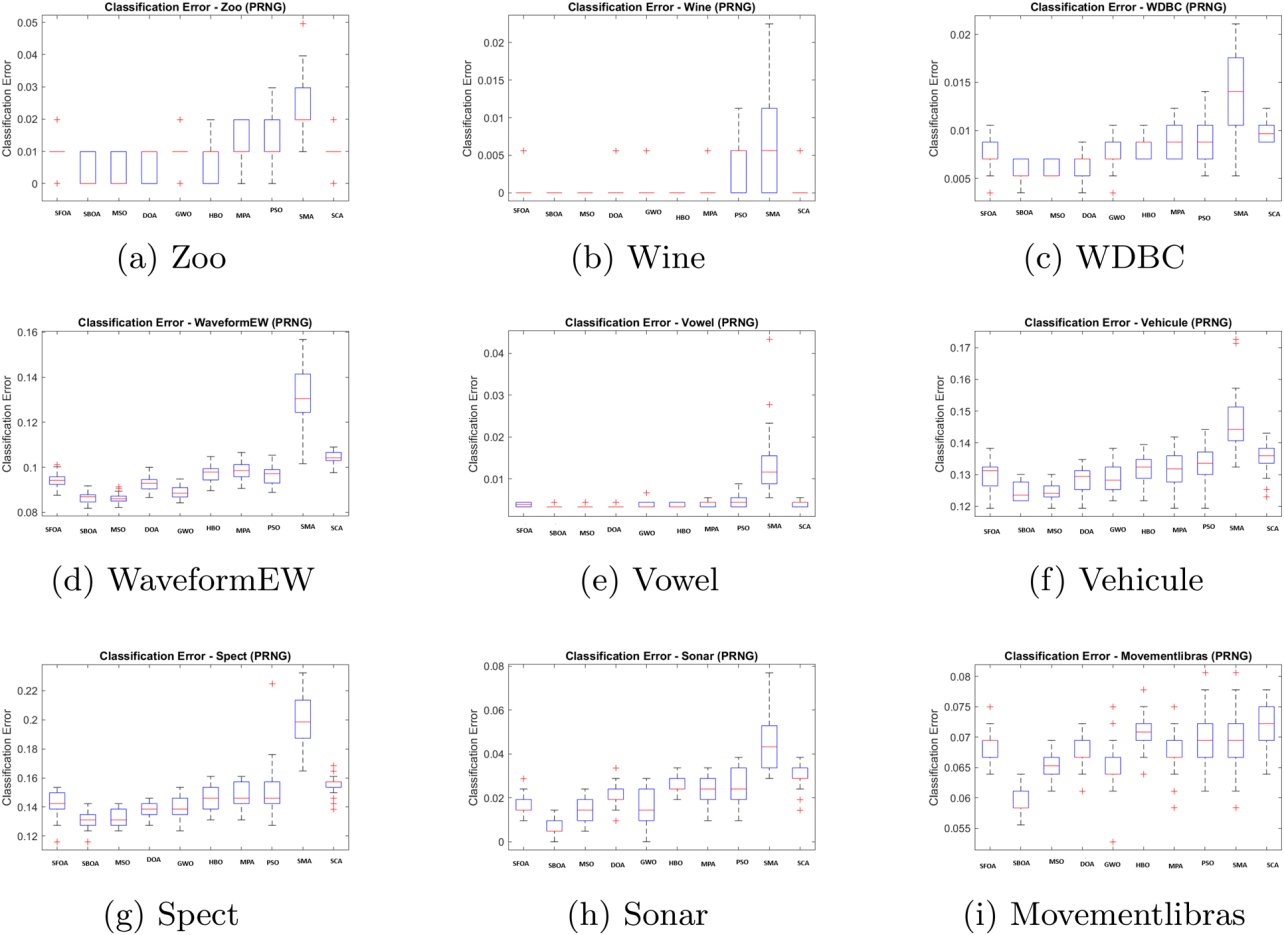
Box plot across datasets (Part 1).

**Fig. 7:**
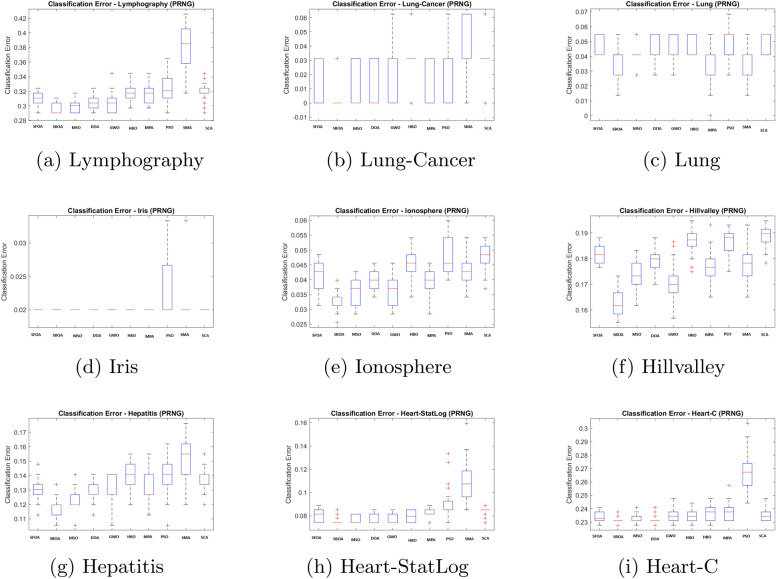
Box plot across datasets (Part 2).

The box plot is used to evaluate the performance of several datasets as a non-parametric measure. However, in descriptive statistics, a box plot represents the groups of numerical data as a graph through their quartets. The maximum or minimum are the largest or lowest data points the algorithm reaches. In these experiments, the box plots for our proposed algorithm compared with the other algorithms over all datasets are presented in Figs. 6,7 and8 .

**Fig. 8:**
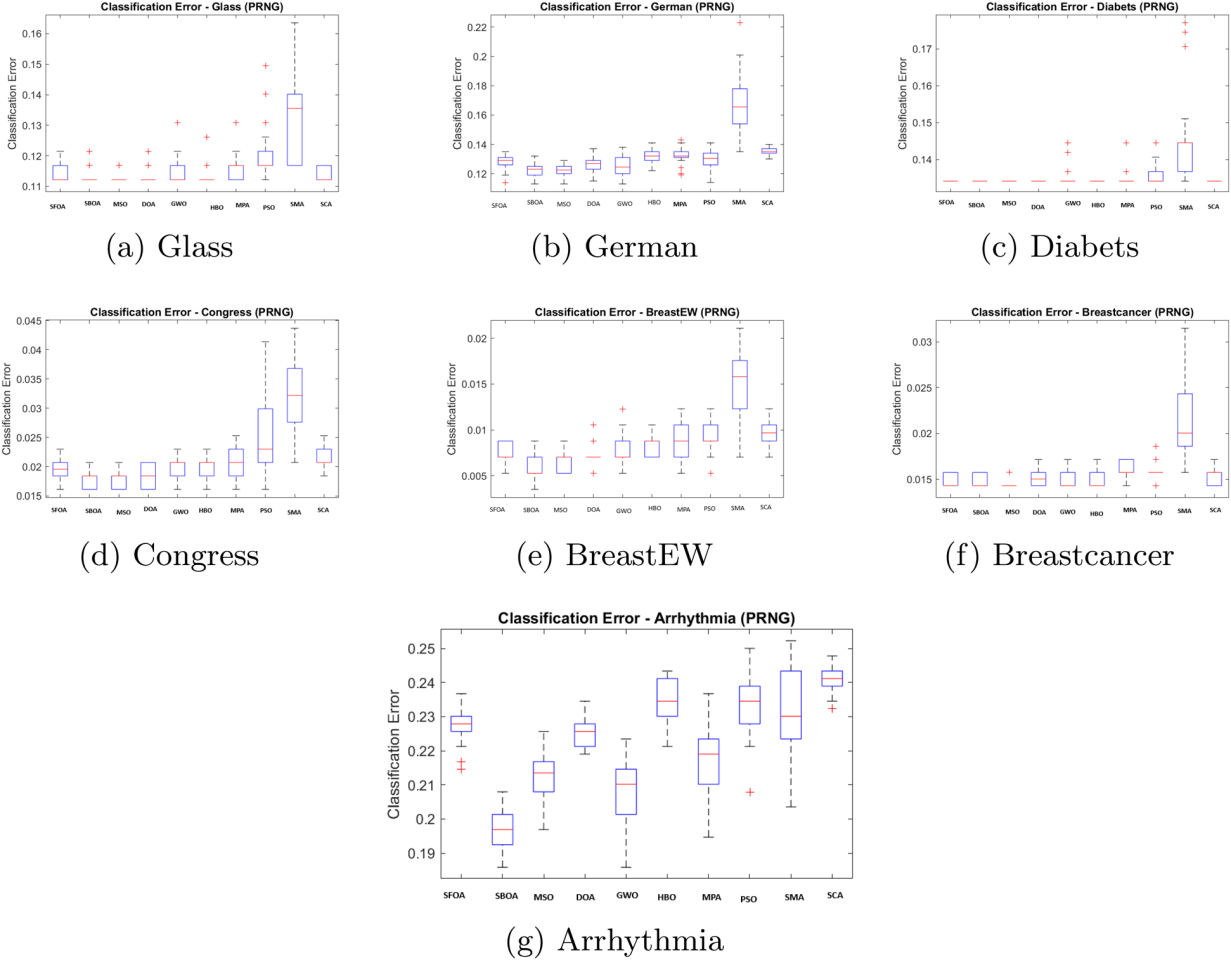
Box plot across datasets (Part 3).

Across the majority datasets, the binary secretary bird optimization algorithm (B-SBOA) demonstrates superior stability and minimal classification error variance, confirming its robust exploration–exploitation balance. Its median classification error consistently falls among the lowest across all algorithms, while maintaining tight interquartile ranges (IQRs) with few or no outliers. This pattern indicates consistent convergence behavior and a reduced tendency to stagnate in local optima, which is attributed to SBOA’s adaptive hunting–escaping phases and the controlled binarization mechanism.

### Wilcoxon rank

To evaluate whether the observed differences between algorithms are statistically effective, the Wilcoxon rank-sum test was applied. This non-parametric test compares the performance of two algorithms at a time across multiple runs without assuming a normal distribution.

A p-value less than 0.05 indicates that the difference between the two algorithms is statistically significant. As shown in Table 9, most p-values are extremely small (e.g., $1\times 10^{-12}$), which confirms that our proposed SBOA has the strongest and most stable performance. SBOA clearly leads with 8 wins, showing the strongest and most consistent performance advantage. This means its superiority is statistically confirmed across many datasets, not by chance.

**Table 9:** Wilcoxon p-values per dataset.

Dataset	SFOA	SBOA	MSO	DOA	GWO	HBO	MPA	PSO	SMA	SCA
Arrhythmia	1.03e−12	1.14e−12	1.14e−12	1.10e−12	1.17e−12	1.13e−12	1.16e−12	1.16e−12	1.19e−12	**1.00e−12**
Breastcancer	4.64e−13	2.90e−13	**1.55e−13**	5.57e−13	3.99e−13	3.99e−13	6.93e−13	5.73e−13	1.13e−12	5.05e−13
BreastEW	7.24e−13	7.11e−13	**3.00e−13**	5.76e−13	6.52e−13	6.75e−13	9.08e−13	8.93e−13	1.11e−12	6.84e−13
Congress	7.87e−13	**4.15e−13**	4.81e−13	7.21e−13	8.25e−13	6.47e−13	1.01e−12	1.06e−12	1.14e−12	7.51e−13
Diabets	**1.69e−14**	**1.69e−14**	**1.69e−14**	**1.69e−14**	**1.64e−14**	**1.69e−14**	6.13e−14	3.85e−13	8.81e−13	**1.69e−14**
German	1.17e−12	1.14e−12	1.16e−12	1.14e−12	1.19e−12	1.16e−12	1.16e−12	1.17e−12	1.20e−12	**1.10e−12**
Glass	2.49e−13	**4.16e−14**	1.18e−13	8.70e−14	3.62e−13	2.01e−13	6.50e−13	8.87e−13	9.95e−13	4.46e−13
Heart-C	8.41e−13	**1.59e−13**	5.94e−13	6.49e−13	1.04e−12	9.27e−13	1.00e−12	1.04e−12	1.16e−12	7.87e−13
Heart-StatLog	9.27e−13	**1.59e−13**	5.47e−13	5.64e−13	4.46e−13	8.93e−13	7.11e−13	8.21e−13	1.16e−12	3.20e−13
Hepatitis	9.24e−13	7.87e−13	**5.51e−13**	7.97e−13	9.43e−13	9.77e−13	1.01e−12	9.86e−13	1.11e−12	8.68e−13

Significant values are in bold.

## Discussion

This study presents the binary version for Secretary Bird Optimization Algorithm SBOA, designed to address both numerical optimization and feature selection tasks effectively. Each population is modified by thresholding random numbers from an RNG. The comparative performance of B-SBOA against other well-established algorithms highlights its efficiency and robustness. From the convergence curve in figs. 3, 4 and 5, it can be noticed that B-SBOA confirms its stability, rapid convergence and a strong exploratory capability, preventing premature convergence and ensuring a broader search space exploration. Analyzing comparative results across 25 benchmark dataset Tables 3, 4, 5, 6, 7 and 8 , shows that the proposed B-SBOA consistently achieved the best or near-best results among all compared algorithms. In most cases, it attained the highest F-score, precision, and recall, reflecting strong classification performance and effective balance between exploration and exploitation. For complex datasets such as Arrhythmia and Hillvalley, SBOA achieved the highest F-score and maintained strong precision–recall balance, demonstrating effective handling of noisy and high-dimensional feature spaces. On the German and Hepatitis datasets, SBOA achieved the best or nearly the best accuracy results, surpassing both swarm-based and physics-inspired methods. This performance highlights the robustness of the algorithm and its ability to generalize well across datasets with different characteristics.

For datasets with clearly separable classes, such as Breastcancer, BreastEW, Congress, and Iris, SBOA achieves very high performance, with F-scores of at least 0.98. The small variation between the maximum, median, and minimum values indicates that the algorithm reaches solutions in a consistent way and converges reliably. The Heart-C and Heart-StatLog datasets show the same trend, with SBOA providing high precision and recall while outperforming alternative methods.

The distribution of classification errors can be clearly observed through the boxplots. SBOA typically shows lower median errors and less variation compared to other approaches, suggesting stable and reliable performance over multiple runs.

For complex datasets like Arrhythmia and Lymphography, SBOA keeps the error distribution compact and avoids extreme results, showing strong robustness to noise and imbalance. In contrast, for structured datasets including Breastcancer, BreastEW, WDBC, and Iris, SBOA achieves very low errors with almost no spread, supporting its repeatable performance.

These findings show that SBOA provides stable and consistent performance in various datasets with different characteristics. By preserving a balanced search strategy, the algorithm effectively controls exploration and exploitation, resulting in robust classification accuracy across all evaluated datasets.

Despite its strong performance, B-SBOA has some limitations. First, the algorithm may face scalability challenges when applied to extremely large datasets with thousands of features. Second, the performance depends on parameter settings such as population size and mutation rate, which may require tuning for different problems. Finally, like most metaheuristic methods, B-SBOA does not guarantee a global optimum, especially in highly complex search spaces.

## Conclusion and future directions

This paper proposed the Binary Secretary Bird Optimization Algorithm (B-SBOA) by implementing thresholding on each solution candidate; to be used in feature selection problems. The algorithm has been experimented on 25 UCI datasets and compared against benchmark metaheuristic algorithms. It was proved that the algorithm performs consistently across various datasets varying from low to high dimensional as well as achieving high accuracy and stable convergence. The algorithm’s ability to balance search strategy allows it to handle noisy and imbalanced data efficiently. These findings suggest that B-SBOA is a practical optimization approach for machine learning applications, particularly in medical and biomedical fields where reliability and interpretability are essential.

Future work might be directed to test the algorithm on a wider range of real-world datasets, such as medical images or financial prediction problems, to verify its generalization ability. Besides, future studies could combine B-SBOA with other metaheuristics in order to form hybrid models that can enhance both exploration and exploitation.

## Data Availability

The datasets used and analyzed during this study are available upon reasonable request from the corresponding authors.
